# Surface assembly and nanofabrication of 1,1,1-tris(mercaptomethyl)heptadecane on Au(111) studied with time-lapse atomic force microscopy

**DOI:** 10.3762/bjnano.5.3

**Published:** 2014-01-09

**Authors:** Tian Tian, Burapol Singhana, Lauren E Englade-Franklin, Xianglin Zhai, T Randall Lee, Jayne C Garno

**Affiliations:** 1 Department of Chemistry, Louisiana State University, 232 Choppin Hall, Baton Rouge, LA 70803, USA; 2 Department of Chemistry and the Texas Center for Superconductivity, University of Houston, Houston, Texas 77204-5003, USA

**Keywords:** liquid AFM, multidentate, nanografting, nanolithography, self-assembly

## Abstract

The solution self-assembly of multidentate organothiols onto Au(111) was studied *in situ* using scanning probe nanolithography and time-lapse atomic force microscopy (AFM). Self-assembled monolayers (SAMs) prepared from dilute solutions of multidentate thiols were found to assemble slowly, requiring more than six hours to generate films. A clean gold substrate was first imaged in ethanolic media using liquid AFM. Next, a 0.01 mM solution of multidentate thiol was injected into the liquid cell. As time progressed, molecular-level details of the surface changes at different time intervals were captured by successive AFM images. Scanning probe based nanofabrication was accomplished using protocols of nanografting and nanoshaving with *n*-alkanethiols and a tridentate molecule, 1,1,1-tris(mercaptomethyl)heptadecane (TMMH). Nanografted patterns of TMMH could be inscribed within *n*-alkanethiol SAMs; however, the molecular packing of the nanopatterns was less homogeneous compared to nanopatterns produced with monothiolates. The multidentate molecules have a more complex assembly pathway than monothiol counterparts, mediated by sequential steps of forming S–Au bonds to the substrate.

## Introduction

Multidentate thiol-based adsorbates attach to gold surfaces through multiple bonds that provide enhanced stability to self-assembled monolayers (SAMs) [1–2]. Although detailed investigations of monodentate thiol-based SAMs have been widely reported, relatively few studies of SAMs derived from bidentate or tridentate thiol adsorbates are available. One might predict that the bulkier headgroups of multidentate adsorbates would strongly influence the kinetics, stability, and surface organization when compared to analogous monodentate *n*-alkanethiol adsorbates. The synthesis of custom-designed multidentate thiol-based adsorbates offers opportunities for generating interfaces having well-defined structure and composition based on either bidentate or tridentate thiol groups, a crosslinked junction, and tailgroups of tunable chemical composition [3–6].

The nature of the headgroup, junctions, hydrocarbon backbone, and tailgroups of SAMs enable designs of complex architectures for applications and surface patterning [7–9]. The stability of organosulfur-based adsorbates on noble metal surfaces is a consideration for applications of SAMs, which impacts the reliability and durability of the related products [10–17]. To realize the full potential of patterning surfaces for manufacturing processes, challenges need to be addressed for designing robust surface coatings that resist damage. Multidentate molecules provide a model surface that will resist self-exchange and surface migration, and enable further steps of chemical reactions with high fidelity. Degradation of alkanethiol SAMs on metal surfaces is caused by UV exposure, thermal desorption, and oxidation [18–19]. It has been reported that SAMs designed with longer chain lengths are more thermally stable than those with shorter chains [19–22]. Multidentate thiols have been investigated as a means to improve the overall stability of alkanethiol SAMs, by forming multiple bonds between a molecule and the surface [2,23]. Several new classes of multidentate alkanethiols have been synthesized that have two or three legs and a binding moiety at each end of the legs [3–6,23]. By appropriate design of the anchoring point, multidentate alkanethiols can be engineered to bind to multiple sites on a noble metal surface. The trend in thermal stability is tridentate alkanethiol > bidentate alkanethiol > *n*-alkanethiol [3,17]. Multidentate adsorbates form stable films that resist desorption and exchange and also resist diffusion across the surface of gold, offering opportunities to generate robust surface nanopatterns.

While the kinetics and mechanisms of film growth of SAMs derived from *n*-alkanethiols have been well-studied [24–28], analogous scanning probe investigations of the surface self-assembly of tridentate alkanethiols on Au(111) have yet to be reported. Within a liquid environment, studies of surface reactions can be accomplished using time-lapse atomic force microscopy (AFM). To understand more completely the surface structure and self-assembly process for multidentate thiols, we chose a tridentate molecule, 1,1,1-tris(mercaptomethyl)heptadecane (TMMH) for in situ AFM studies. The orientation of TMMH on the surface was investigated using approaches with liquid imaging and scanning probe lithography. By using a liquid sample cell for AFM studies, fresh reagents can be introduced to the system for monitoring step-wise changes of a surface over time, such as before and after nanofabrication steps. Side-by-side comparisons of the surface structures of multidentate adsorbates versus *n*-alkanethiol SAMs were accomplished using nanografting to give a local measurement of film thickness, referencing the well-known dimensions of *n*-alkanethiols as a baseline.

## Results and Discussion

Liquid environments expand the capabilities for scanning probe protocols to provide insight for dynamic processes at the nanoscale [29]. For example, studies of the elastic modulus of SAMS and protein films was accomplished in liquid media using force modulation AFM [30]. Liquid imaging often has advantages for AFM studies, particularly for conducting in situ investigations of chemical or biochemical reactions. Liquid media has benefits for improving resolution, since the amount of force applied between the tip and sample can be reduced [31]. Surface changes after immersion in different liquids can be investigated using time-lapse AFM imaging. Investigations of surfaces throughout the course of chemical self-assembly reactions have been monitored with AFM in liquid media [27]. Further, by injecting new molecules into the sample cell, AFM-based nanofabrication can be accomplished using protocols of nanoshaving and nanografting [32–33]. Of course, the solvents chosen for AFM liquid experiments should be optically transparent, and must have a relatively slow rate of evaporation (e.g., water, ethanol, butanol, or hexadecane).

**Figure 1:**
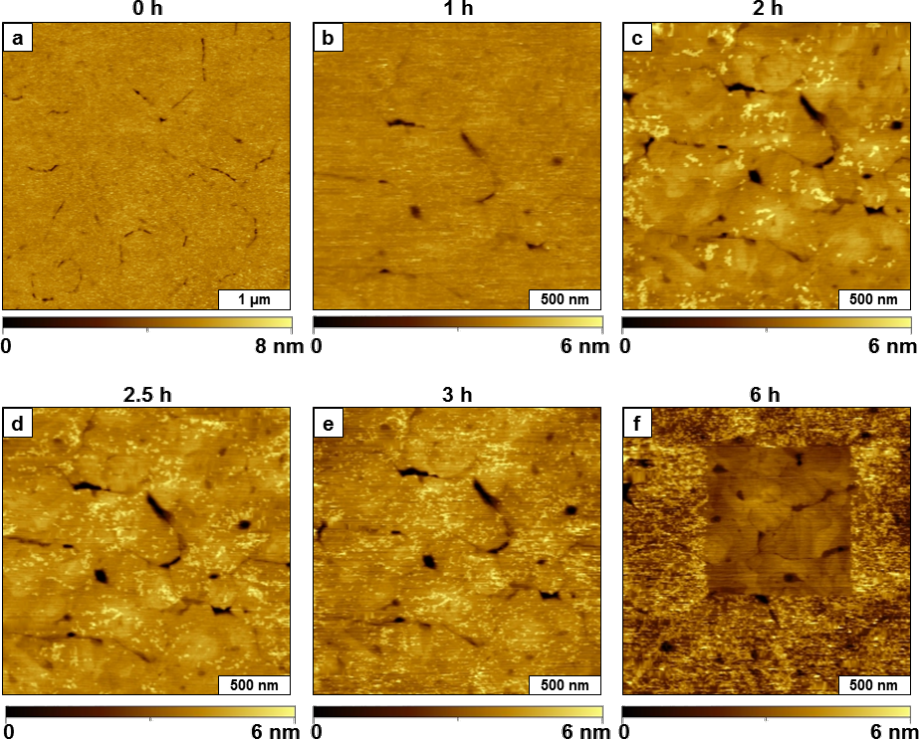
Solution self-assembly of TMMH on gold viewed by time-lapse AFM. Topography images (contact-mode in liquid) acquired after (a) 0 h; (b) 1 h; (c) 2 h; (d) 2.5 h; (e) 3 h; (f) 6 h after injection of TMMH solution.

**Surface self-assembly of TMMH**. A liquid AFM study was accomplished using time-lapse imaging to investigate the self-assembly of TMMH molecules on template-stripped gold (Figure 1). The surface was imaged in ethanol before injecting the TMMH solution (Figure 1a). The image reveals relatively flat domains bordered by several cracks and scars; the sites of the defects furnish reference landmarks for in situ imaging. After injecting a solution of TMMH in ethanol (0.01 mM) into the liquid cell, small changes were observed during the first hour. At this concentration, a few adsorbates became apparent after 1 h (Figure 1b). Increases in surface coverage were detected as time progressed. Time-lapse images after 2, 2.5, and 3 h are presented in Figure 1c–e with a distinct arrangement of surface landmarks to anchor the location for acquiring successive images. However, as the surface coverage of TMMH increased, the landmarks became indistinguishable (Lateral force images corresponding to the topography frames of Figure 1 are provided in the Supporting Information File 1, Figures S1, S2, and S3). To continue the experiment, a square region was shaved clean to provide a reference location for further time points (Figures S3 and S4 in the Supporting Information File 1). For nanoshaving, a higher force was applied to the AFM tip during scans to sweep away TMMH molecules from the gold surface (Figure 1e). The experiment was terminated after 6 h before the surface reached saturation coverage (Figure 1f).

**Figure 2:**
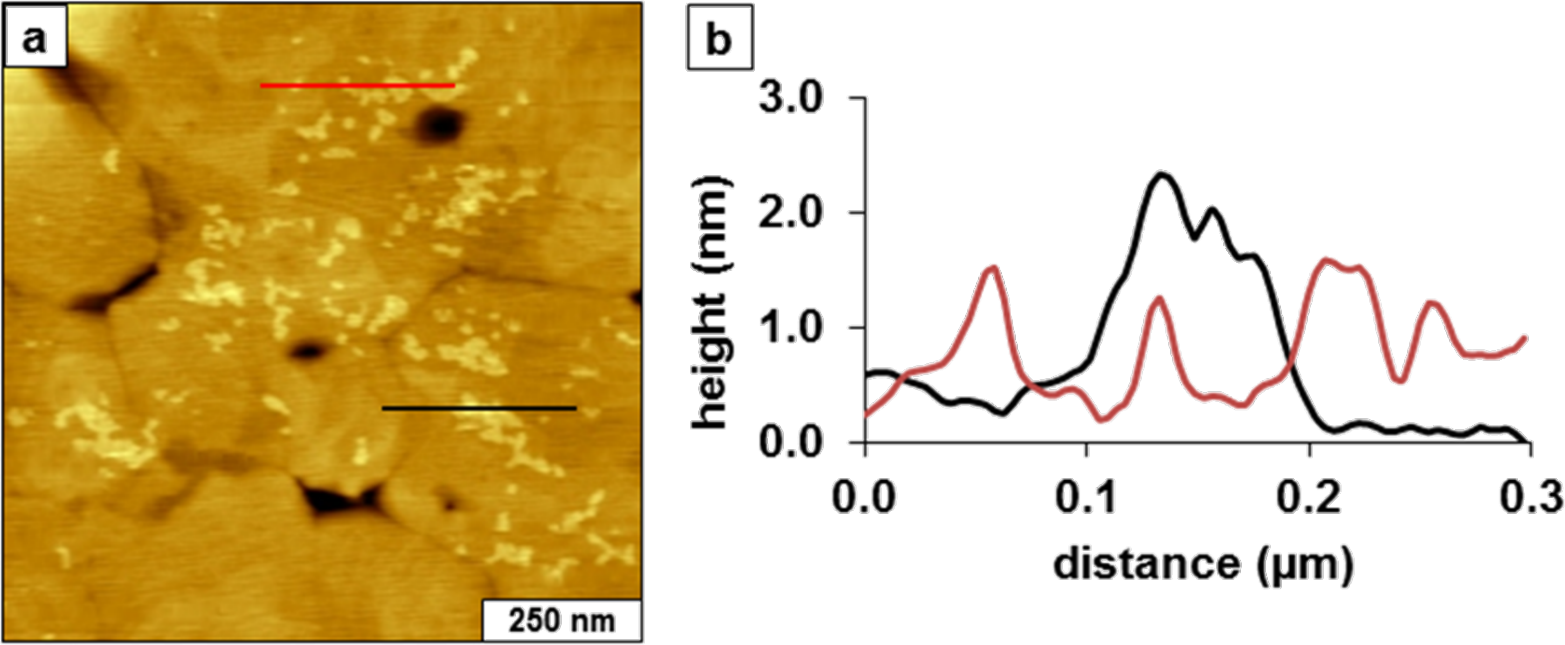
Representative cursor profiles of the side-on and standing phases of TMMH measured after 2 h of immersion.

With higher magnification, the thickness of the adsorbates can be measured more precisely (Figure 2). The initial bright structures (Figure 2a) appear to attach preferentially to the edges of gold terraces; at this magnification, however, it is difficult to clearly resolve the smallest adsorbates. There are multiple overlapping terraces throughout the areas of the substrate, so evidence of a mobile phase is not conclusive. Several heights are apparent for the adsorbates ranging from 0.5 to 2.2 nm. The shortest structures correspond approximately to the thickness of an alkyl chain with a side-on orientation. The 0.5 nm measurement concurs with the height expected for a physisorbed phase with the backbone of the molecule oriented parallel to the substrate [27]. The tallest heights measured 2.2 nm, and this thickness corresponds to a standing upright configuration of the TMMH, which has a theoretical length of 2.3 nm. A distribution of intermediate heights ranging from 0.8 to 2.0 nm were measured for the adsorbates in Figure 2, which suggests a complex self-assembly pathway for TMMH.

When considering a possible surface assembly model for the observations of Figure 1 and Figure 2, it appears that the initial orientation of the molecule is arranged with a side-on configuration, with the alkyl backbone aligned parallel to the substrate. It is likely that one sulfur of the tridentate molecule attaches to the surface in the initial molecular adsorption step. As time progresses, a second sulfur attached to the surface with rearrangement to a canted orientation, in which the backbone is lifted from the surface to adopt a tilted configuration. The adsorbates with thickness values between 0.8 and 2.0 nm correspond to the transition from a lying-down phase. Over a longer time interval, eventually the molecule rearranges to an upright orientation (2.2 nm), which likely has all three sulfur groups attached to the surface. Although we have no direct evidence of the numbers of sulfur groups attached to the substrate using AFM characterizations, the range of intermediate height measurements of Figure 2 suggest a step-wise attachment of the sulfur moieties.

**Figure 3:**
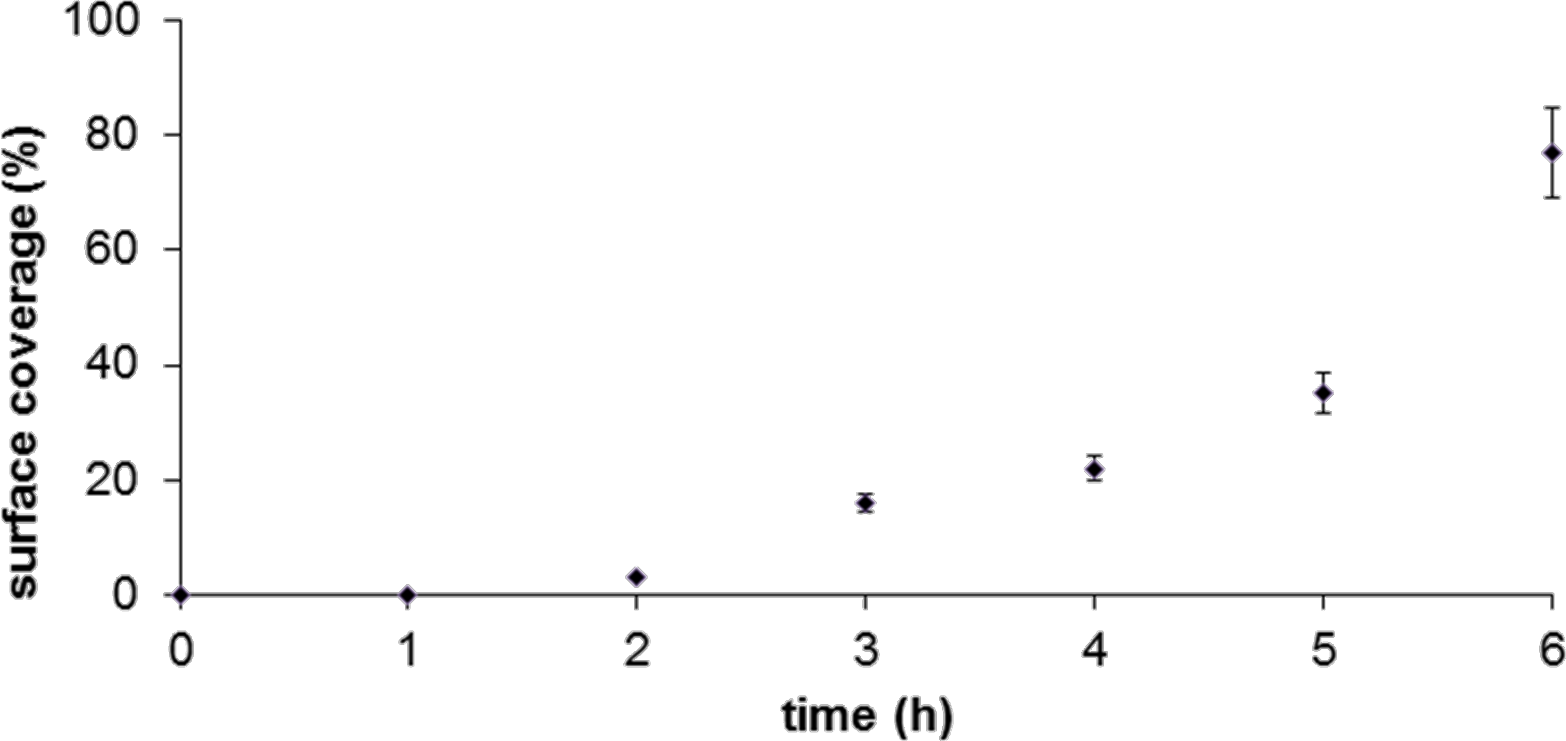
Gradual increase in surface coverage of the taller (standing) phase of TMMH as time progressed.

Kinetic trends for the surface-assembly of the taller phase of TMMH are plotted in Figure 3. The binding of TMMH is relatively slow at this concentration. At higher concentrations, multilayers of TMMH were formed through dithiol bonds; therefore dilute conditions were used to slow the rate of surface deposition [27]. As shown by the surface coverage estimates in Figure 3, the rate of surface adsorption of TMMH increased after 2 h, suggesting that interactions between neighboring molecules as surface density increased influenced the rate of surface attachment. The data for Figure 3 were constructed from analyzing the surface area of regions containing TMMH adsorbates and are a composite of lying-down, standing and multilayer adsorbates. After TMMH bound to surface sites, molecules began to associate and attach to the surface more quickly. Incomplete monolayers were observed for brief immersion steps, and mature, densely packed SAMs were formed after at least 24 h immersion. The initial studies with tridentate TMMH molecules reveal slow adsorption >6 h to reach a standing configuration with dilute conditions of 0.01 mM solution.

**Figure 4:**
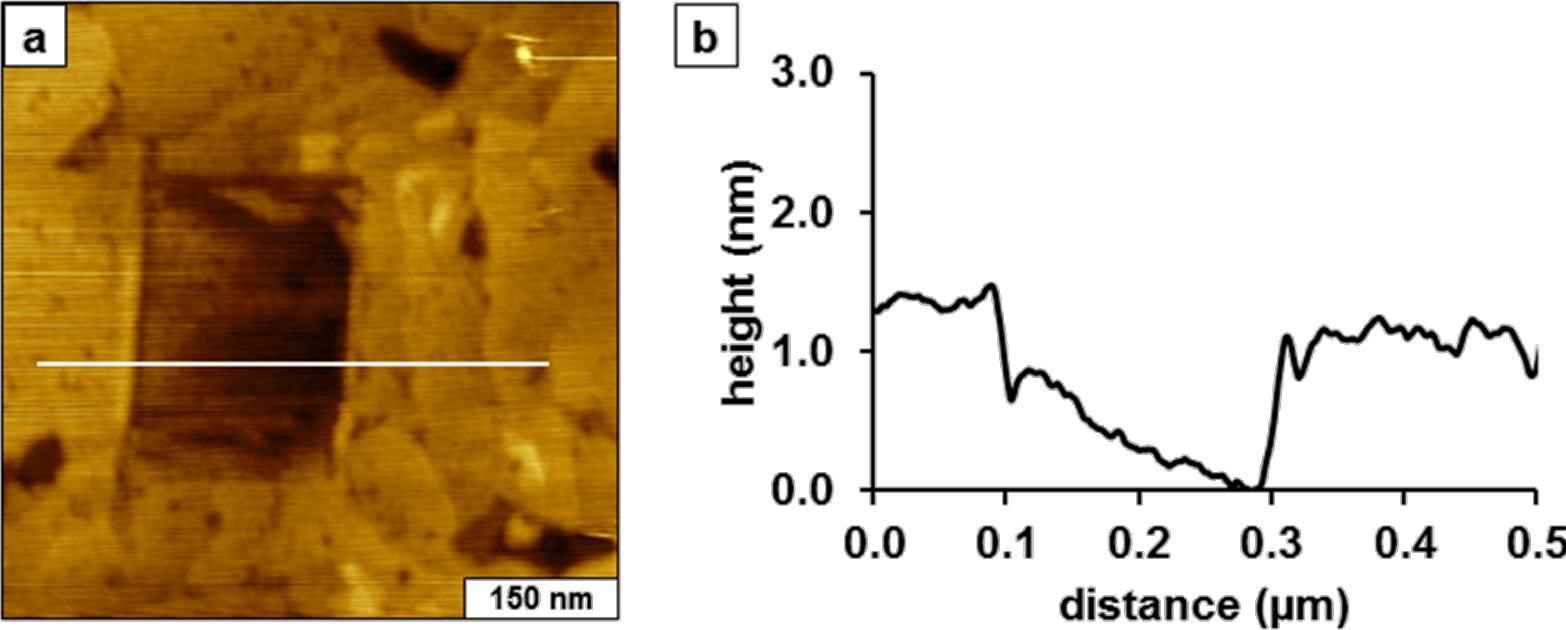
Nanoshaved square within a SAM of TMMH. (a) Topography image acquired in ethanol; (b) Line profile across the square pattern.

**Nanoshaving of a TMMH film on gold**. A convenient way to measure locally the thickness of an organothiol film with liquid AFM is to shave away a small area of the film by applying a higher force to the AFM probe and sweeping. An example of nanoshaving is shown in Figure 4 for a 200 × 200 nm^2^ area of gold that was uncovered by the AFM tip. Some of the molecules are deposited at the left and right sides of the nanopattern, indicated by the bright edges. However, most of the molecules dissolved in the liquid media or are swept away by the scanning action of the AFM tip. A possible concern when increasing the force to the AFM tip is that the probe might become dull or break. However, for this example the tip retains its sharpness because the pinhole defects and contours of the step edges of the underlying gold beneath the SAM of TMMH (Figure 4a) can be resolved, even after the tip was used for fabrication steps. In comparison to the example of nanoshaving in Figure 1f, the SAM is more densely packed after 30 h immersion in TMMH (see Figure 4). The thickness of the SAM is 1.0 ± 0.2 nm measured at the right edge of the nanopattern. The baseline within the nanoshaved area has a slope due to the nature of the substrate. The left side has a hill of adsorbates from the material scraped to the side by the nanoshaving process and is unreliable for measuring the thickness.

**Nanografting of *n*-alkanethiols within TMMH.** By injecting new molecules into the sample cell, AFM-based nanofabrication can be accomplished using nanoshaving and nanografting protocols [32–33]. Approaches with nanolithography enable side-by-side comparisons of the surface structures of multidentate adsorbates versus *n*-alkanethiol monolayers (i.e., film thickness, morphology). Our experimental strategies rely on using a liquid sample cell for AFM studies, since fresh reagents can be introduced to the system, and stepwise changes of the surface before and after nanofabrication can be monitored in situ. For experiments in liquid media, the method of surface nanografting developed by Xu et al. was used to inscribe nanopatterns [33]. For these experiments, *n*-alkanethiol SAMs provided an internal calibration tool; essentially, the well-known dimensions of *n*-alkanethiol monolayers furnish an in situ ruler for local measurements of the thickness of molecular films [7,34–36].

Our protocols for nanografting used either dodecanethiol or TMMH as matrix SAMs that were prepared by immersion in ethanolic solutions. Areas of the matrix were selected for nanoshaving or nanografting of patterns to enable a side-by-side comparison of molecular thickness. The steps of experiments were captured with AFM images before and after fabricating nanopatterns within a liquid environment. The same AFM probe was used for writing nanopatterns and for in situ sample characterizations.

**Figure 5:**
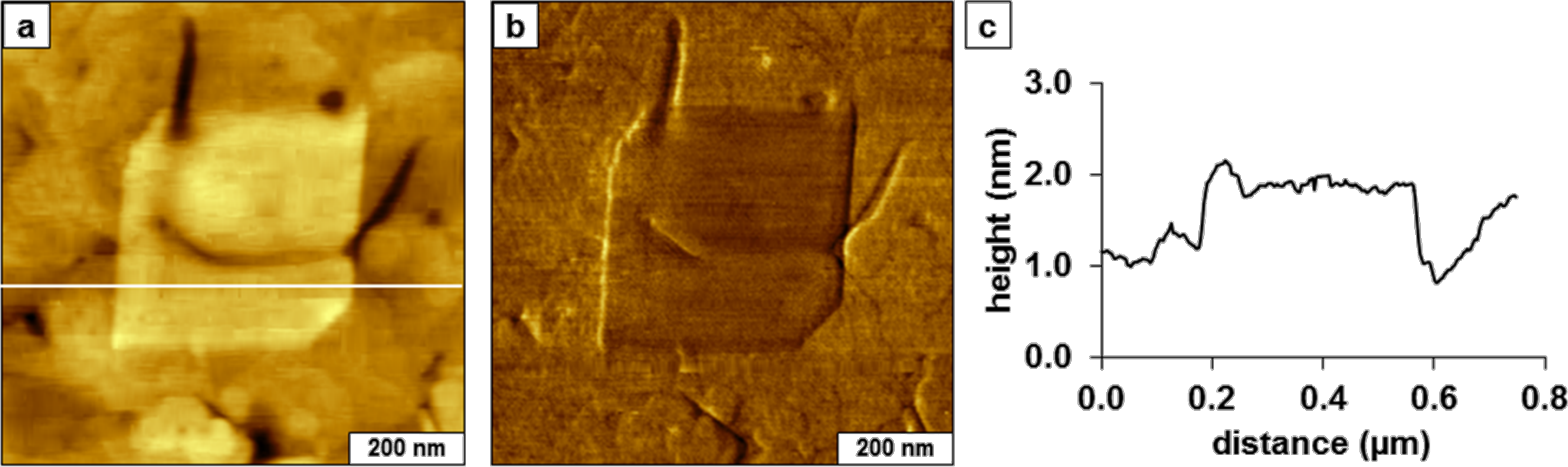
Nanografting of octadecanethiol (ODT) within a densely-packed TMMH matrix. (a) Topography image acquired in contact mode; (b) corresponding lateral force image. (c) Height profile taken across the square pattern in (a).

A square pattern of octadecanethiol (ODT) was nanografted into a matrix of TMMH, as shown in Figure 5. The bright square consists of densely-packed alkanethiols with methyl-terminated headgroups (Figure 5a). A slightly darker contrast is observed for the nanografted pattern compared to the matrix for the lateral force image of Figure 5b, even though TMMH and ODT are both terminated with methyl groups. The darker contrast could be attributable to differences in packing density: the nanografted pattern appears to be more dense than the surrounding SAM of TMMH, which is consistent with observations from previous studies of tridentate SAMs on gold [19]. The surrounding areas of the TMMH matrix are shorter than ODT. The expected thickness of an octadecanethiol SAM on gold is 2.2 nm, and the octadecanethiol square is approximately 1 nm taller than the TMMH matrix (Figure 5c). Thus, for this example the local thickness of TMMH measures 1.2 ± 0.2 nm.

**Figure 6:**
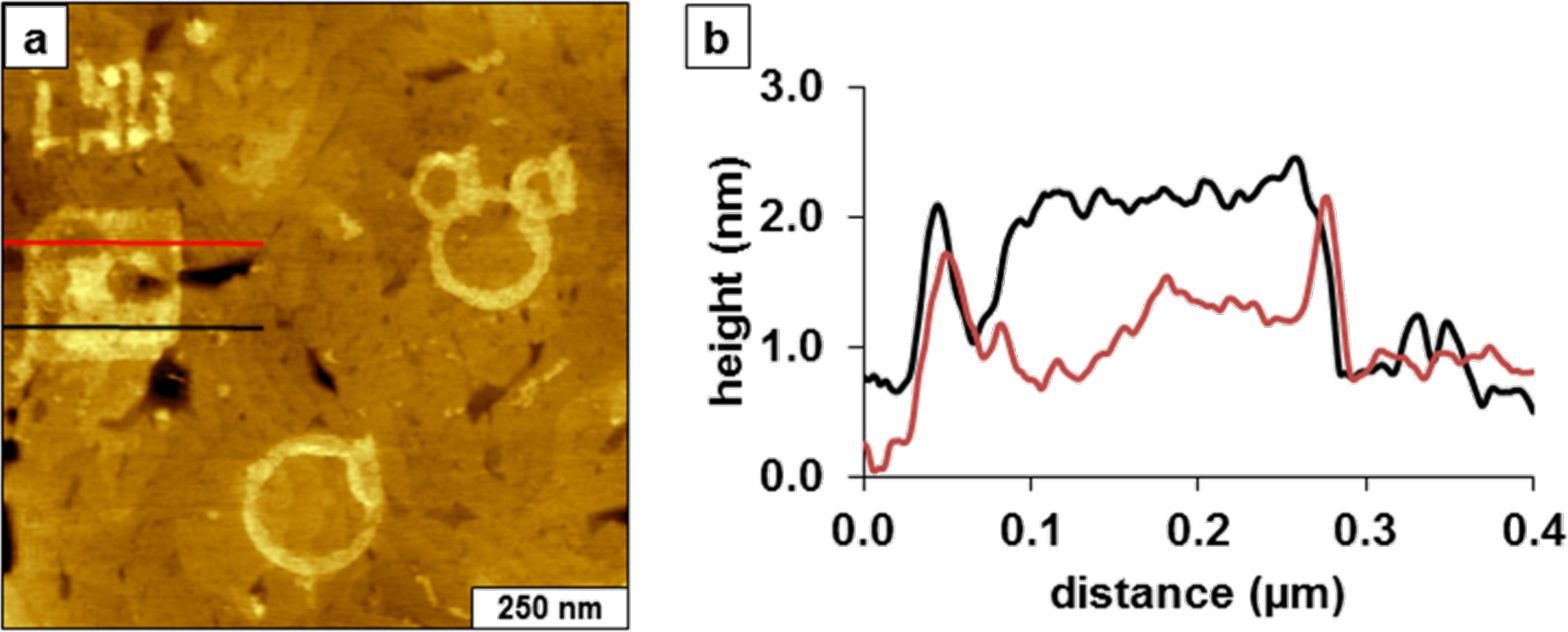
Nanografting of 11-mercaptoundecanoic acid (MUA) within a matrix of TMMH. (a) Topography view of multiple patterns that were nanografted within an 800 × 800 nm^2^ region. (b) Cursor profiles across the terrace square of (a).

To acquire additional local measurements of the thickness of TMMH films, nanopatterns of 11-mercaptoundecanoic acid (MUA) were grafted within a matrix of TMMH (Figure 6a). Each of the patterns shown in Figure 6a were inscribed by multiple sweeps across the same selected region, which produced a double-layer thickness for the circles and letter shapes. It has previously been reported that multiple sweeps during nanografting of carboxylic acid-terminated SAMs produced bilayer nanopatterns [36]. The square nanopattern of MUA on the left side of the topography frame measures 200 × 200 nm^2^, and reveals a two-tier design with single- and double-layer thickness. Cursor lines were drawn across the top and bottom areas of the MUA nanopatterns (Figure 6b) measuring 0.5 ± 0.2 and 2.0 ± 0.2 nm above the TMMH matrix for the single and double layers, respectively. The profile across the monolayer region of the pattern (Figure 6b, red line) measuring ~0.5 nm above the matrix indicates that the SAM derived from TMMH is ~1 nm thick. The areas of the pattern with a double layer (Figure 6b, black line) are 2 ± 0.2 nm taller than the TMMH matrix. Since a double layer of MUA would be 3.0 nm thick, this analysis likewise indicates a height of ~1 nm for the SAM derived from TMMH.

**Figure 7:**
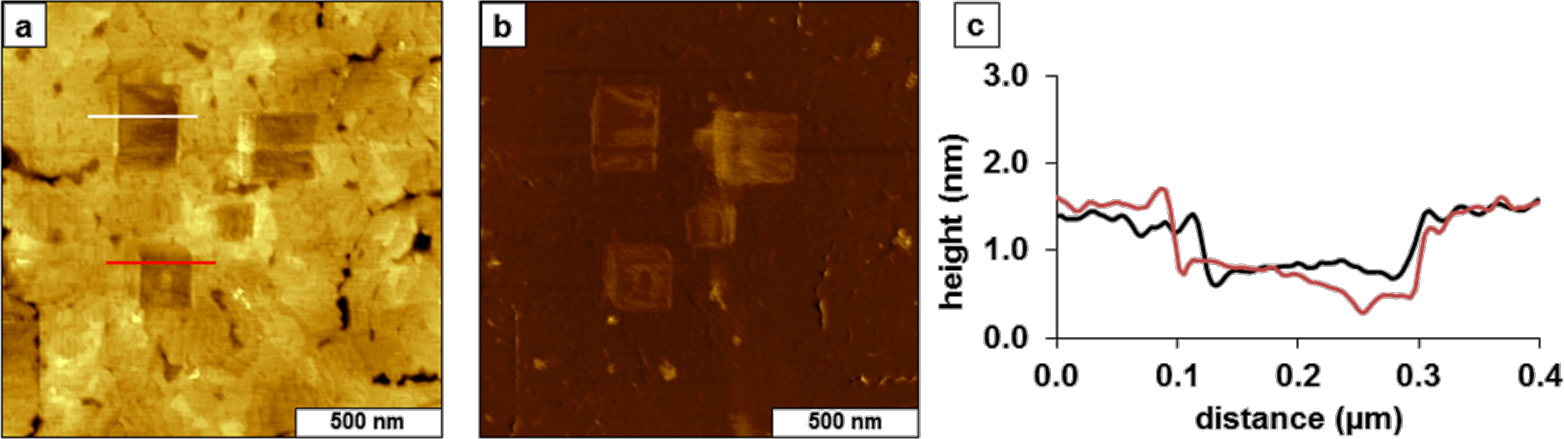
Nanografted patterns of TMMH within a dodecanethiol SAM. (a) Topograph of squares of TMMH (1.5 × 1.5 µm^2^); (b) lateral force image for (a); (c) height profile across two TMMH nanopatterns in (a**)**.

Further experiments were conducted with nanografting of TMMH nanopatterns within a methyl-terminated dodecanethiol SAM (Figure 7). The dodecanethiol SAM was prepared from 1 mM ethanolic solution, and the TMMH nanografted patterns were prepared with 0.01 mM solution. The expected thickness of a dodecanethiol SAM is 1.5 nm, as a reference for evaluating the thickness of TMMH nanostructures. Four nanopatterns were written within the methyl-terminated SAM (Figure 7a,b). The height of the TMMH squares is shorter than the surrounding matrix SAM of dodecanethiol. The difference in thickness ranges from 0.6–0.9 nm, which corresponds to a thickness of 0.7 ± 0.3 nm for nanografted patterns of TMMH (Figure 7c). The simultaneously acquired lateral force image (Figure 7b) reveals the edges of the nanopatterns as well as the step edges of the underlying gold substrates. The surface density of TMMH within the nanografted regions is not homogeneous; for example, the top right square seems to have a greater density of TMMH than the patterns on the left side of the frame. The pattern at the top right side has patches of brighter and darker shades, which correspondingly have different thickness measurements within the nanofabricated area. Further experiments are planned to evaluate how the physical parameters for nanofabrication (line speed, line density) influence the thickness of TMMH patterns.

When nanografting *n*-alkanethiols, the molecules attach to the gold surfaces directly in a standing-up configuration due to the effects of spatial confinement [37]. However, the tridentate molecules have a larger headgroup, which influences the packing density [19]. The thickness values derived from each of the different AFM experiments are summarized in Table 1, and are in fair agreement for measurements at the nanoscale.

**Table 1:** Thickness measurements of TMMH on gold substrates.

AFM protocol	TMMH^a^ thickness (nm)	Example

Time-lapse AFM study, upright adsorbates on gold	1.0 ± 0.2	Figure 2
Nanoshaving of mature SAM of TMMH	1.0 ± 0.2	Figure 4
Nanografted ODT within TMMH matrix SAM	1.2 ± 0.2	Figure 5
Nanografted MUA within TMMH matrix SAM	1.0 ± 0.2	Figure 6
Nanografted TMMH within dodecanethiol SAM	0.7 ± 0.3	Figure 7

^a^The error is estimated to be at least 0.2 nm from the thickness of a gold step.

Unlike our earlier observations from unconstrained surface assembly (Figure 1) that several hours were required for TMMH to bind to gold surfaces to form a SAM, nanografting experiments disclosed that TMMH attached immediately following the scanning track of the AFM tip (Figure 7). However, the shorter height suggests a less-dense packing arrangement for the nanografted patterns of TMMH with the bigger foot (i.e., larger molecule). The height of nanografted patterns is shorter than that expected for an upright configuration of TMMH, likely attributable to the dilute conditions of the experiment. This may be attributable to an incomplete surface assembly of all three sulfurs of the tridentate group, with only one or two sulfur atoms attaching to the substrate during nanografting protocols.

For the nanografting experiments with TMMH as the matrix monolayer, the overall film thickness indicates a tilted configuration. Using the value of 1.0–1.2 nm as the thickness of a mature TMMH SAM from Table 1, the heptadecane backbone would be tilted ~59–64° with respect to surface normal, compared to the well-known 30° tilt of *n*-alkanethiol SAMs. The interplay of a wider intermolecular spacing between adjacent backbones and the larger geometry of the tridentate “foot” provide the rationale for a less dense arrangement of TMMH films. The tridentate adsorbates formed a monolayer in which the alkyl chains are highly disordered on the surface as compared to SAMs derived from monodentate *n*-alkanethiols reported from studies with sum frequency generation imaging microscopy [38]. The packing density followed the trend monodentate > bidentate > tridentate. There is a possibility that only one or two of the sulfur groups bind to the substrate which would likewise contribute to a tilted orientation for TMMH. Previous studies of the thermal stability of tridentate SAMs show increased stability for tridentate alkanethiols compared to *n*-alkanethiols [3,17]; thus for our model we propose that three sulfurs are anchored to the substrate. In future experiments, we plan to evaluate the parameters of concentration and solvents for producing SAMs of TMMH, and will investigate the stability of multidentate films with exposure to heat, UV-irradiation and oxidation.

## Conclusion

Using dilute ethanolic solutions, the surface self-assembly of TMMH onto Au(111) was imaged with time-lapse AFM for 6 h. With higher concentration, multilayers of TMMH were produced. Protocols of nanografting and nanoshaving were used to compare the heights of TMMH with *n*-alkanethiol SAMs using side-by-side AFM views. The films of TMMH formed from relatively dilute conditions (0.01 mM) were less densely packed than for *n*-alkanethiol SAMs that were prepared at mM concentration.

## Experimental

**Scheme 1:**
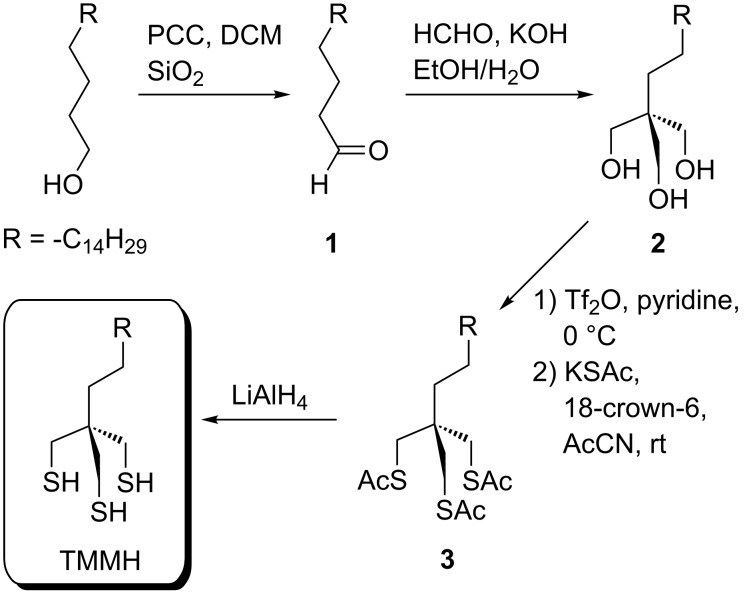
Strategy used to prepare 1,1,1-tris(mercaptomethyl)heptadecane (TMMH).

**Materials and reagents.** Ethanol (200 proof) was obtained from AAper Alcohol and Chemical Co. (Shelbyville, KY). Flame-annealed gold films on mica substrates (150 nm thickness) were obtained from Agilent Technologies (Phoenix, AZ). Template-stripped gold films were prepared on glass slides using Epotek 377, as previously described by Wagner et al [39]. Octadecanethiol and dodecanethiol were purchased from Sigma Aldrich (St. Louis, MO) and used as received. The tridentate molecule 1,1,1-tris(mercaptomethyl)heptadecane (TMMH) was synthesized as described below and is illustrated in Scheme 1 in a similar manner as previously reported [5,40].

For the synthetic procedures, all organic solvents were dried with calcium hydride (CaH_2_) and distillated before use. Pyridinium chlorochromate (PCC) and lithium aluminum hydride (LiAlH_4_) were purchased from Alfa Aesar. 1-Octadecanol (ReagentPlus^®^, 99%), formaldehyde (37 wt % in H_2_O), trifluoromethanesulfonic anhydride (≥99%), 18-crown-6 (≥99.9%), pyridine (anhydrous, 99.8%), potassium thioacetate (98%), and anhydrous acetonitrile (AcCN) were purchased from Sigma-Aldrich. All other reagents were used without further purification.

**Octadecanal (1)*.*** The aldehyde was synthesized by using a modification of literature methods [5,41]. Specifically, pyridinium chlorochromate (26.89 g, 124.7 mmol) and silica gel (30 mg) were mixed and suspended in 100 mL of dry CH_2_Cl_2_. The solution of 1-octadecanol (20.13 g, 74.42 mmol) in CH_2_Cl_2_ was added into the stirred mixture. Stirring was continued for 4 h at rt, and the black chromium compounds were removed by passage through a short pad of silica gel. The filtrate was concentrated to dryness and purified by column chromatography on silica gel, eluting with 4% diethyl ether in hexanes to afford octadecanal **1** (16.25 g, 60.53 mmol, 81%). ^1^H NMR (500 MHz, CDCl_3_) δ 0.88 (t, *J* = 7.0 Hz, 3H, C*H*_3_), 1.20–1.36 (m, 28 H), 1.59–1.66 (m, 2H, C*H*_2_CH_2_CHO), 2.42 (td, *J* = 1.9, 7.4 Hz, 2H, C*H*_2_CHO), 9.76 (t, *J* = 1.9 Hz, 1H, C*H*O).

**1,1,1-Tris(hydroxymethyl)heptadecane (2)** [42]. Octadecanal (10.25 g, 38.18 mmol) and aqueous formaldehyde (37 wt % in H_2_O, 30 mL, excess) were dissolved in 60 mL of aqueous ethanol (50%). To this stirred mixture was added a solution of potassium hydroxide (3.59 g, 64.0 mmol) in 60 mL of aqueous ethanol (50%). The reaction mixture was stirred for 4 h at rt and heated to 60 °C for 6 h. The ethanol was removed by rotary evaporation, and the residue was extracted with diethyl ether (3 × 100 mL). The combined organic phases were washed with water (3 × 100 mL), dried over MgSO_4_, and concentrated to dryness. The crude products were purified by column chromatography on silica gel, eluting with 4% methanol in CH_2_Cl_2_ to give a white solid (4.05 g, 12.3 mmol, 32%). ^1^H NMR (500 MHz, CDCl_3_) δ 0.88 (t, *J* = 7.0 Hz, 3H, C*H*_3_), 1.10–1.33 (m, 30H), 2.53 (t, *J* = 5.1 Hz, 3H, 3O*H*), 3.74 (d, *J* = 4.7 Hz, 6H, 3C*H*_2_OH).

**1,1,1,-Tris(acetylthiomethyl)heptadecane (3).** Pyridine (30 mL, 0.49 mol) was added to a solution of triol **2** (3.89 g, 11.8 mmol) in dry CH_2_Cl_2_ (50 mL) at 0 °C and stirred for 15 min. Afterward, trifluoromethanesulfonic anhydride (15 mL, 94 mmol; Tf_2_O) in dry and cold CH_2_Cl_2_ (30 mL) was added dropwise to the reaction solution over a period of 20 min. The reaction mixture was then stirred at 0 °C for 4 h. The mixture was diluted with CH_2_Cl_2_ (150 mL), washed with 2 M HCl and 5% NaHCO_3_, and dried with MgSO_4_. The solvent was removed under reduced pressure to give the crude tritriflate (5.25 g). This intermediate was used without further purification in the next step. A solution of crude tritriflate (5.25 g), 18-crown-6 (24.88 g, 94.15 mmol), and potassium thioacetate (10.75 g, 94.15 mmol) in anhydrous acetonitrile (120 mL) was stirred at rt for 8 h. The resulting precipitate was removed by filtration, and the filtrate washed with 5% NaCl (300 mL) and dried with MgSO_4_. The organic phase was concentrated in vacuo. The residue was purified by column chromatography on silica gel, eluting with hexanes/ethyl acetate (7:1) to afford **3** (4.86 g, 9.63 mmol, 82% yield). ^1^H NMR (500 MHz, CDCl_3_) δ 0.87 (t, *J* = 7.0 Hz, 3H), 1.18–1.35 (m, 30H), 2.34 (s, 9H, CH_2_SC(O)C*H*_3_), 2.98 (s, 6H, C*H*_2_SC(O)CH_3_).

**1,1,1-Tri(mercaptomethyl)heptadecane (TMMH)**. A solution of **3** (2.80 g, 5.55 mmol) in dry THF (80 mL) was added dropwise to a suspension of LiAlH_4_ (1.26 g, 33.3 mmol) in dry THF (60 mL). The mixture was stirred at rt for 6 h and then quenched with H_2_O and acidified with 2 M HCl under argon (H_2_O and 2 M HCl were degassed by bubbling with N_2_ gas before use). After stirring for 10 min, the mixture was extracted with CH_2_Cl_2_ (3 × 100 mL). The combined organic layers were washed with H_2_O and brine. After drying the solution with Na_2_SO_4_, the solvent was removed by rotary evaporation, and the resulting residue was chromatographed on silica gel with hexanes/ethyl acetate (3:1) to afford TMMH (1.60 g, 4.22 mmol, 76% yield). ^1^H NMR (500 MHz, CDCl_3_) δ 0.88 (t, *J* = 7.0 Hz, 3H), 1.17 (t, *J* = 8.7 Hz, 3H), 1.21–1.33 (m, 28H), 1.35–1.41 (m, 2H), 2.56–2.60 (m, 6H, 3C*H*_2_SH); ^13^C NMR (125 MHz, CDCl_3_) δ 14.13, 22.71, 23.27, 29.05, 29.27, 29.36, 29.53, 29.68, 30.04, 30.09, 31.94, 32.42, 41.39.

**Atomic force microscopy.** Either a model 5500 or 5420 scanning probe microscope (Agilent Technologies, Chandler, AZ) equipped with PicoView v1.8 software was used for the AFM characterizations and scanning probe lithography. Images were acquired using contact mode in a liquid cell, which can hold up to 1 mL of solution. Imaging and fabrication were accomplished with silicon nitride tips, which had an average spring constant of 0.5 N/m (Bruker Instruments, Camarillo, CA). Digital images were processed and analyzed with Gwyddion v.2.25 software [43]. Analysis of surface coverage was accomplished by manually selecting a threshold value to convert images to black and white data sets, and counting pixels using the UTHSCSA *ImageTool* program (developed at the University of Texas Health Science Center at San Antonio, Texas and available from the Internet by anonymous FTP from maxrad6.uthscsa.edu).

**AFM study of the self-assembly of TMMH from solution.** A piece of template-stripped gold on glass was placed in the liquid cell and imaged continuously. Initially, the sample was imaged in ethanolic media to obtain a representative view of the gold substrate. Next, a solution of TMMH (0.01 mM) in ethanol was injected into the liquid cell to monitor the growth of TMMH in situ. A relatively low concentration of TMMH (0.01 mM) was selected to enable surface assembly at a sufficiently slow rate to enable monitoring with time-lapse AFM images. After introducing TMMH solution into the sample cell, images were acquired every 15 min for 3 h for the same area. The liquid cell was replenished with fresh TMMH solution at 90 minute intervals because the ethanol evaporates over time. After 3 h, the tip was moved for imaging a new area to minimize the effects of perturbing the surface by the scanning probe. Images were acquired at 30 min intervals during the later stages of the experiment.

**Scanning probe lithography (nanoshaving and nanografting).** Nanoshaving experiments were accomplished by applying a high force (2–5 nN) to sweep a selected area ten times with 256 lines/frame in ethanolic media. The nanoshaved patterns could be imaged in situ using the same probe by returning to a lower force setting. Nanografting experiments were accomplished by sweeping an area under high force in a liquid cell containing an ethanolic solution of the molecule to be patterned. Multiple cursor profiles were acquired for measuring the thickness of nanopatterns. The error term was estimated to be at least the height of a monatomic gold step (0.2 nm). Solutions of either octadecanethiol or dodecanethiol solutions were prepared at a concentration of 1 mM for nanografting. A dodecanethiol SAM was prepared by immersing a piece of template-stripped gold in a 1 mM ethanolic solution for 12 h. A monolayer film of TMMH was prepared by immersing a piece of template-stripped gold in a 0.01 mM ethanolic solution for 72 h. A lower concentration was used for TMMH to prevent forming multilayer films.

## Supporting Information

Additional AFM images are provided that include lateral force frames and images acquired at other selected time points during surface self-assembly (Figures S1, S2, S3 and S4).

File 1 Additional AFM images.
