# Supplementary material for: Surface assembly and nanofabrication of 1,1,1-tris(mercaptomethyl)heptadecane on Au(111) studied with time-lapse atomic force microscopy
Source: Beilstein J Nanotechnol. 2014 Jan 9;5:26–35. doi: 10.3762/bjnano.5.3 (PMC3896263; doi:10.3762/bjnano.5.3)
Supplement: File 1 — Additional AFM images. [file Beilstein_J_Nanotechnol-05-26-s001.pdf]

Supporting Information

for

Surface assembly and nanofabrication of 1,1,1-tris(mercaptomethyl)heptadecane on Au(111) studied with time-lapse atomic force microscopy

Tian Tian[1], Burapol Singhana[2], Lauren E. Englade-Franklin[1], Xianglin Zhai[1], T. Randall Lee[2], and Jayne C. Garno[1]*[§]

Address: [1] Department of Chemistry, Louisiana State University, 232 Choppin Hall, Baton Rouge, LA 70803, USA and [2] Department of Chemistry and the Texas Center for Superconductivity, University of Houston, Houston, Texas 77204-5003, USA

*Corresponding Author

E-mail: Jayne C. Garno - jgarno@lsu.edu
[§]Fax: 225-578-3458

Additional AFM images

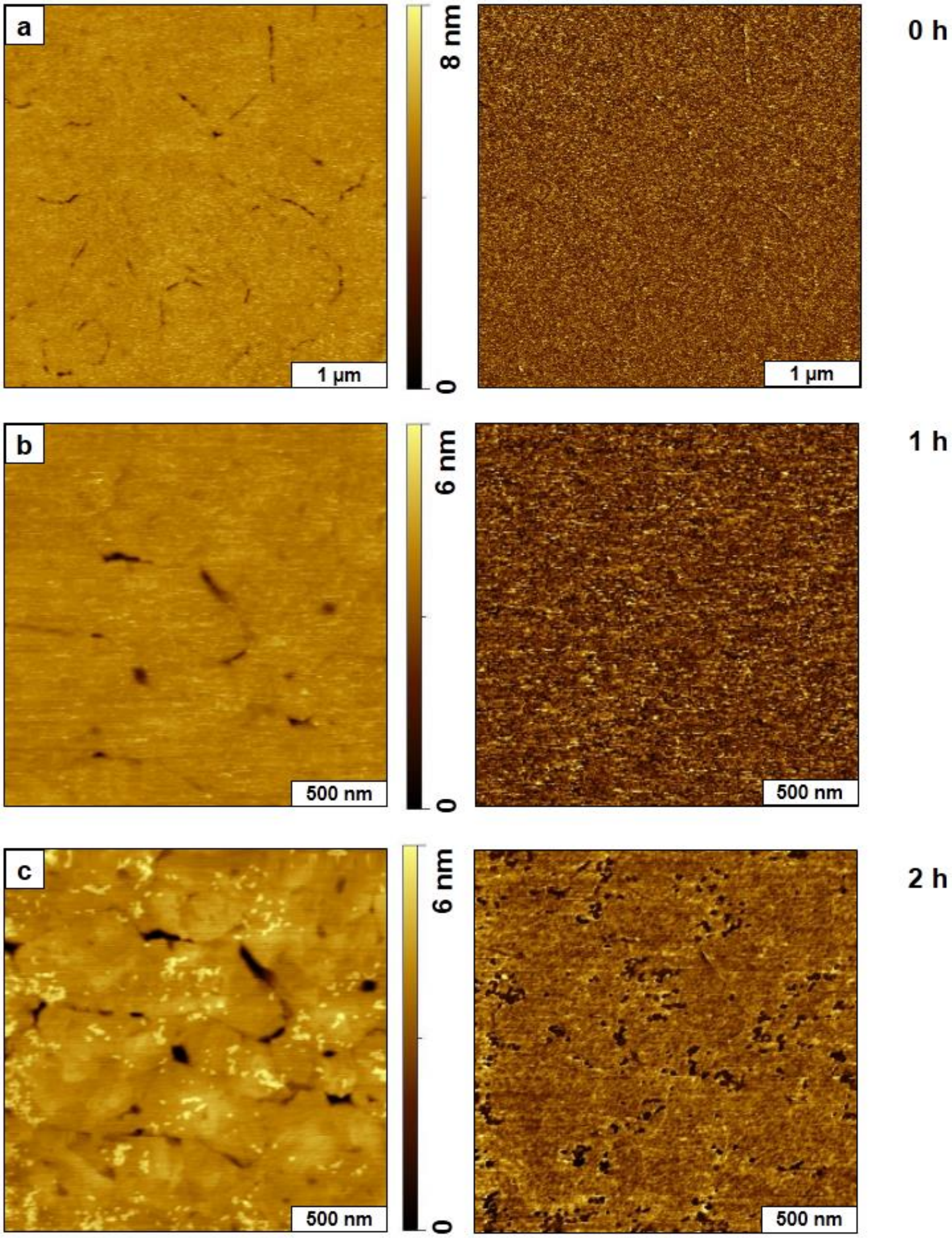

Figure S1: The interactions between the AFM tip and the different phases of TMMH molecules on the gold surface are disclosed by lateral force frames for the topography images of Figure 1. Topography (left) and corresponding lateral force images (right) of the solution phase self-assembly of TMMH on a gold surface at selected immersion intervals: (a) 0 h; (b) 1 h; (c) 2 h.

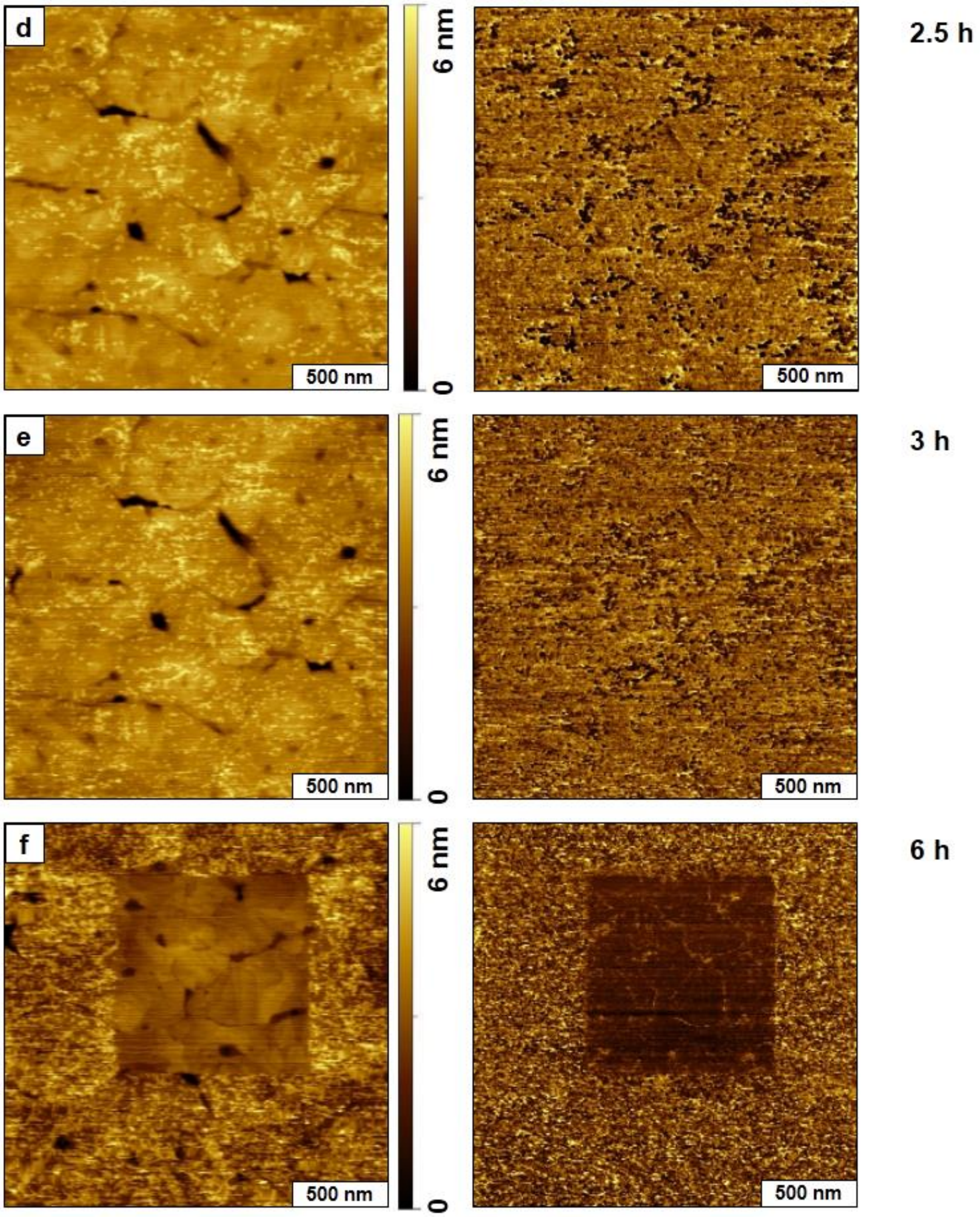

Figure S2: Lateral force images are shown for the topography images of Figure 1. Topography (left) and corresponding lateral force images (right) of solution phase self-assembly of TMMH on a gold surface after selected immersion intervals: (d) 2.5 h; (e) 3 h; (f) 6 h. The images correspond to the same time points shown in Figure 1.

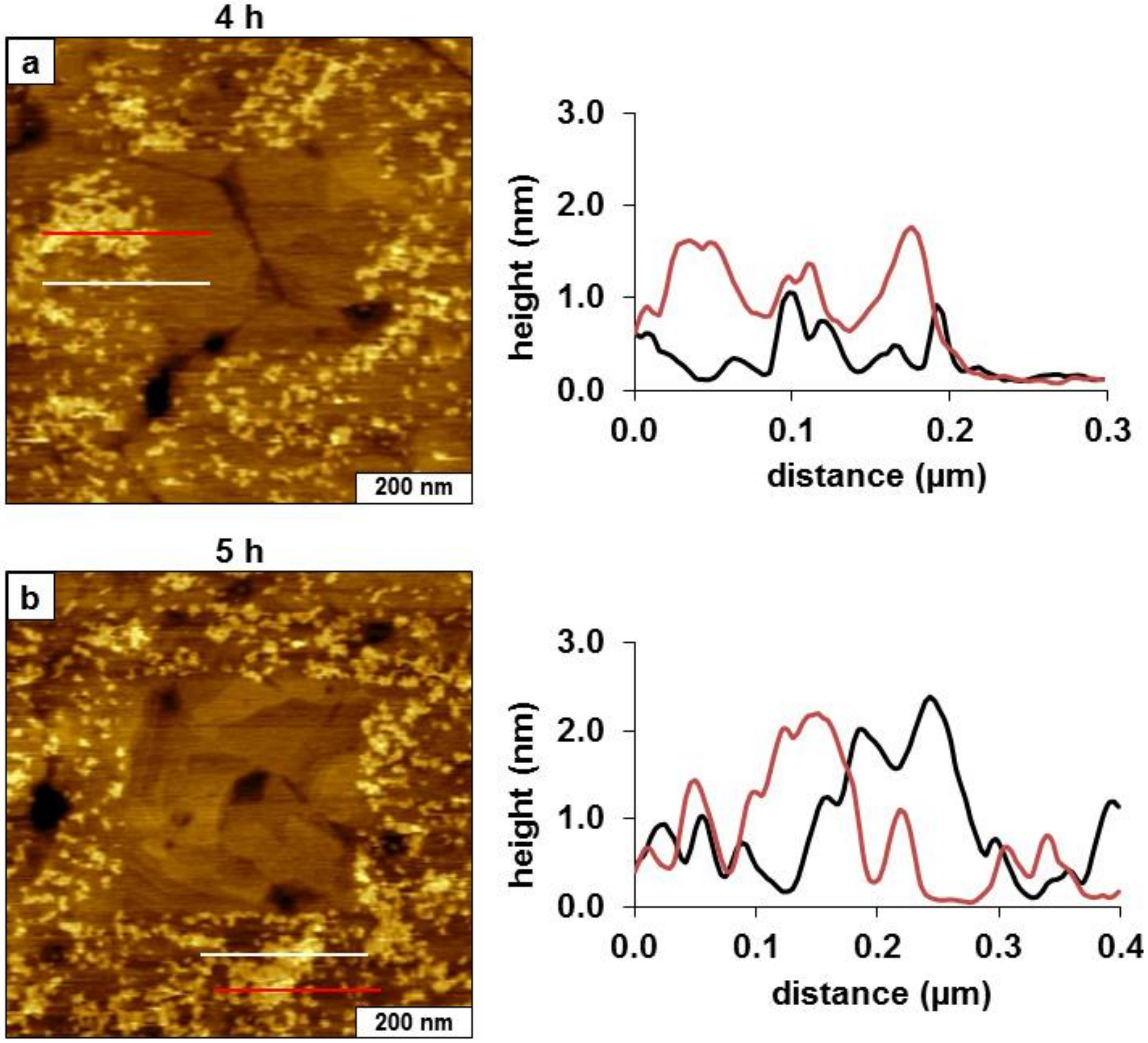

Figure S3: Snapshots at later time points of the solution self-assembly of TMMH for areas of gold, with nanoshaved reference sites. Contact mode AFM topographs acquired after (a) 4 h; and (b) 5 h of immersion in a 0.01 mM solution of TMMH in ethanol.

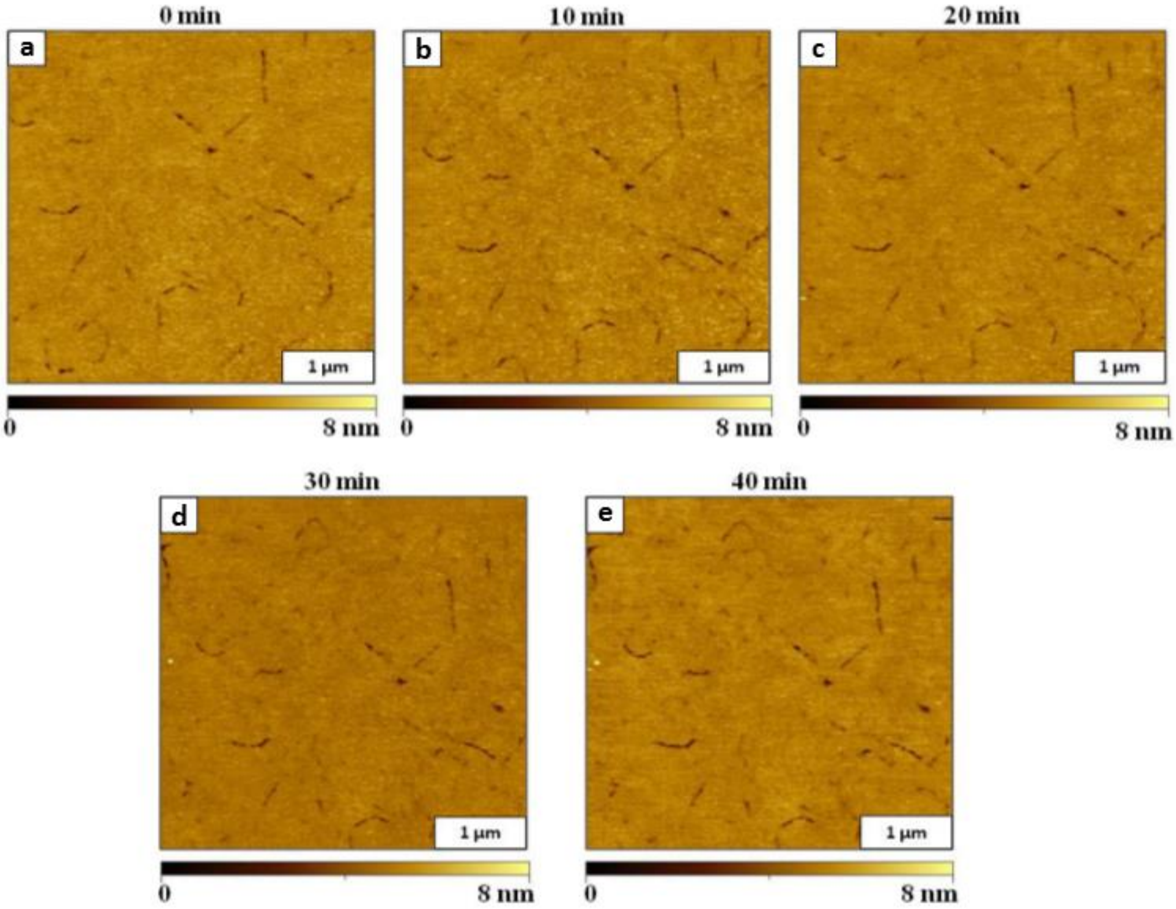

Figure S4: Early snapshots of the gold surface after introducing the TMMH solution (0.01 mM in ethanol). Topography images acquired at (a) 0 min (b) 10 min; (c) 20 min; (d) 30 min; and (e) 40 min of exposure to 0.01 mM TMMH solution.
